# Potential role of epithelial–mesenchymal transition induced by periodontal pathogens in oral cancer

**DOI:** 10.1111/jcmm.18064

**Published:** 2023-11-30

**Authors:** Yiwei Ma, Yingyi Yu, Yuqing Yin, Liu Wang, Huishun Yang, Shiyin Luo, Qifan Zheng, Yaping Pan, Dongmei Zhang

**Affiliations:** ^1^ Department of Periodontics, School of Stomatology China Medical University Shenyang China; ^2^ Department of Periodontics and Oral Biology, School of Stomatology China Medical University Shenyang China

**Keywords:** epithelial–mesenchymal transition, *Fusobacterium nucleatum*, oral cancer, periodontal pathogens, *Porphyromonas gingivalis*

## Abstract

With the increasing incidence of oral cancer in the world, it has become a hotspot to explore the pathogenesis and prevention of oral cancer. It has been proved there is a strong link between periodontal pathogens and oral cancer. However, the specific molecular and cellular pathogenic mechanisms remain to be further elucidated. Emerging evidence suggests that periodontal pathogens‐induced epithelial–mesenchymal transition (EMT) is closely related to the progression of oral cancer. Cells undergoing EMT showed increased motility, aggressiveness and stemness, which provide a pro‐tumour environment and promote malignant metastasis of oral cancer. Plenty of studies proposed periodontal pathogens promote carcinogenesis via EMT. In the current review, we discussed the association between the development of oral cancer and periodontal pathogens, and summarized various mechanisms of EMT caused by periodontal pathogens, which are supposed to play an important role in oral cancer, to provide targets for future research in the fight against oral cancer.

## 1 INTRODUCTION

Microorganisms found in the oral cavity are among the most abundant in the human body, and they play a crucial role in maintaining a healthy oral physiological environment. Therefore, imbalances between microbiome and susceptible individuals can lead to oral and systemic diseases. Periodontitis is just a common inflammatory disease caused by dysbiosis. It is proved by accumulated evidence that periodontitis influences the initiation and/or development of a variety of systemic diseases. On the contrary, an increased incidence of oral abnormalities including periodontitis was reported to be related to systemic disease.^1^ For the past few years, the scientific community has recognized extensively on links between periodontal pathogens and cancer. For instance, some periodontal pathogens, namely *Porphyromonas gingivalis* (*P. gingivalis*), *Prevotella intermedia* and *Tannerella forsythia* were found to increase the risk of gastrointestinal cancer.^2^ Additionally, oral microbiome‐associated primary tumours can be observed in the stomach, oesophagus, pancreas, colon and rectum and especially in the oral cavity.^2^

Head and neck cancer is the sixth most common malignancy worldwide according to statistics, and as reported, oral squamous cell carcinoma (OSCC) is the most ubiquitous.^3^ To date, the major recognized risk factors of oral cancer include tobacco use, alcohol consumption, betel quid chewing, inappropriate dietary habits and poor oral hygiene.^4^ However, 15%–20% of the patients with oral cancer are non‐smokers or non‐alcohol drinkers or do not have any of the above unhealthy habits.^5^ Therefore, it is necessary to explore other potential pathogenic mechanisms of oral cancer. Indeed, previous researches have already proved that there is a strong link between periodontal disease and oral cancer. According to a meta‐analysis, oral cancer is more likely to occur in patients with periodontal disease.^6^ Additionally, some epidemiological researches have already reported an obvious positive relationship between periodontal disease and oral cancers,^7^ especially OSCC.^8^ As recognized periodontal pathogens, the roles of *P. gingivalis* and *Fusobacterium nucleatum* (*F. nucleatum*) in the development of oral cancer have also been studied in recent years. Periodontal pathogens have been suggested as a possible etiological factor for oral cancer, independent of smoking and drinking. However, the impact of periodontal pathogens on cancer is multifaceted, and the specific molecular and cellular pathogenic mechanisms remain to be further studied. In recent research, E‐cadherin levels decreased significantly while vimentin levels increased in OSCC cells treated with heat‐killed *P. gingivalis* or *F. nucleatum* after 8 days, suggesting epithelial–mesenchymal transition (EMT) has occurred.^9^

EMT is a cellular process in that epithelial cells lose typical epithelial characteristics and transdifferentiate into cells with mesenchymal features. Normally, according to the different biological context in which EMT occurs, researchers divide it into three subtypes. Type 1 EMT is associated with embryogenesis and organ development. Type 2 EMT is involved in wound healing, tissue regeneration and organ fibrosis.^10^, ^11^ When the injury is moderate and acute, the healing event caused by type 2 EMT is considered as reparative fibrosis, but in ongoing chronic inflammation, it leads to persistent fibrosis and ultimately to organ parenchymal destruction.^12^ Meanwhile, type 3 EMT occurs during cancer progression.^10^, ^11^ It is reported that after undergoing EMT, cancer cells are characterized by increased motility, invasion, stemness and resistance to drugs and apoptosis.^13^, ^14^ Up to now, EMT has been demonstrated in numerous primary cancers, such as colorectal,^15^ breast,^16^ pancreatic,^17^ lung^18^ and head and neck.^19^ The research on EMT has shown an explosive growth in recent years, but the understanding of this complex cell biological program may still be very partial. On the one hand, a large number of core or other EMT‐TFs, signalling pathways, and noncoding RNAs, can drive EMT either directly or indirectly. On the other hand, in various biological contexts, the diversity of EMT phenotypic manifestations is becoming more apparent. This means that the definition and interpretation of EMT may differ in different research areas in specific contexts.^20^ Notably, EMT is not a binary process in cancer progression, for cancer cells are capable of expressing different levels of both epithelial and mesenchymal markers, which is referred to as partial, incomplete or hybrid EMT states.^21^, ^22^ There is even a view that cells which undergo partial EMT, instead of complete EMT, are more likely to metastatically spread.^22^

EMT makes a significant impact on the malignant metastasis of oral cancer as well. With the promotion of EMT, oral cancer cells shed from the primary site, metastasize through blood vessels or the lymphatic system, and eventually proliferate into new nidus.^23^ This metastatic property of EMT‐conferring cancer cells increases the risk of recurrence and decreases the survival rate in oral cancer.^24^ Also, EMT provides an extracellular environment around the tumour that encourages cancer cells' survival and escape from the immune system, resulting in resistance to various chemotherapeutic agents.^25^ In a word, EMT plays an essential role in oral cancer progression.

As a newly discovered risk factor for oral cancer, periodontal pathogens have been found to induce EMT process, which has attracted our attention. Accumulated evidence shows periodontal pathogens‐induced EMT promotes carcinogenesis and cancer development, but the biological molecular mechanisms are still not fully understood.

Here, a comprehensive literature search was carried out in MEDLINE, PubMed, Web of Science and Google Scholar from the establishment of the database to June 2023, and the search terms included the following medical subject headings and free words: ‘epithelial–mesenchymal transition’; ‘EMT’; ‘oral cancer’; ‘periodontitis’; ‘periodontal disease’; ‘EMT‐TFs’; ‘microRNA’; ‘lncRNA’; ‘periodontal pathogens’; ‘*Porphyromonas gingivalis*’; and ‘*Fusobacterium nucleatum*’. All possible articles, whether original studies or reviews, were considered for this review, and there were no restrictions on the date of publication and journal. The language was limited to English. Moreover, we also searched the reference lists of included articles. We reviewed the evidence supporting the involvement of EMT in oral cancer. Then the evidence linking periodontal pathogens and oral cancer were discussed. The core machinery responsible for EMT caused by periodontal pathogens was summarized, with the aim to better understanding the specific molecular and cellular pathogenic mechanisms of periodontal pathogens in oral cancer and providing targets for future research in the fight against oral cancer.

## 2 PERIODONTAL PATHOGENS AND ORAL CANCER

### 2.1 Periodontal pathogens can be found in clinical samples of oral cancer

Initially, the association of periodontal pathogens with oral cancer was derived from the comparison of the microbial species present in tumour tissues with that in non‐tumorous materials. In 1998, Nagy et al. reported for the first time that higher levels of *P. gingivalis* and *F. nucleatum* were found in OSCC tissues based on bacteria culture and biochemical identification, but this study was only limited to the tumour surface of OSCC.^26^ With the improvement of sequencing technology, next‐generation sequencing was widely used to characterize the composition of microbial communities, especially those species have very low abundance. Using 16S rRNA gene sequencing, Al‐hebshi et al. compared epithelial swabs from tumour patients and healthy subjects.^27^ The results showed many microbial species were found to differ in abundance between the cases and controls, with *Fusobacterium* having the highest frequency among OSCC samples.^27^ Similarly, Zhang et al. found the abundance of 10 bacteria, including *F. nucleatum*, in the tumour site on buccal mucosal of OSCC patients was higher than that of opposite normal tissues.^28^ Chang and her colleagues performed FISH on tissue slices and found *P. gingivalis* in cancer tissues were significantly more abundant than in paracancarcinoma and normal tissues, which widely existed in the epithelium and deep layer of tumours.^29^ A metatranscriptomic analysis of the oral microbiota associated with OSCC sites in humans was performed, in order to determine whether microbial metabolic activities change in tumour environment. The conclusion was only *F. nucleatum* was highly active in OSCC tissues, with a number of metabolic activities including proteolysis, cobalamin biosynthesis and iron ion transport being overrepresented.^30^

Noticeably, the results of different studies are not completely consistent. On the one hand, this may be due to the variety of methods employed in these studies in terms of the techniques used for microbiota analysis, the type of clinical sample and the choice of control group. On the other hand, according to Al‐hebshi's research, the association of microbiota with OSCC was at the functional level instead of composition level.^27^ That means it is not a few specific bacteria contributing to OSCC, but rather some of their bacterial functions.^27^

In addition, a study also showed that compared with normal gingival tissue, a higher level of *P. gingivalis*‐specific staining can be detected in gingival squamous cell carcinoma (GSCC), especially in poorly‐differentiated gingival carcinoma specimens.^31^ Another study, comparing microbiome of the GSCC with periodontitis microbiome, found *Fusobacterium* was more abundant in cancerous tissues.^32^ Generally, multiple researches exploiting clinic samples have indicated a significant increase in the abundance of periodontal pathogens despite different sources of tumour tissues and different locations of detection.

### 2.2 Infection of periodontal pathogens promotes oral cancer in animal models

Kamarajan et al. employed mouse floor‐of‐mouth models to simulate human OSCC. They injected OSCC cells infected with *P. gingivalis*/*F. nucleatum* into mice, and found these mice showed greater tumour burden compared with the mice injected with nonpathogen infected cells.^33^ Similarly, Gallimidi et al. reported the establishment of a mouse model of chronic periodontitis combined with oral carcinoma, which incorporated *P. gingivalis*/*F. nucleatum* infection with an oral carcerogen, 4‐nitroquinoline‐1‐oxide (4NQO).^34^ Periodontal pathogens may interact directly with cancerous and precancerous oral epithelial cells via activation of Toll‐like receptors (TLR) and eventually induce important effectors driving OSCC growth and invasiveness.^34^ Another significant alteration during 4NQO‐induced carcinogenesis is the level and profile of serum fatty acid, which is further aggravated by *P. gingivalis* infection.^35^ *P. gingivalis* can upregulate the expression of two essential enzymes in the synthesis of fatty acids (FASN and ACC1), and participate in the pathway of de novo fatty acid synthesis to change the lipid metabolism in oral cancer.^35^ Moreover, *P. gingivalis* is able to trigger immunoevasion of OSCC by protecting cancer cells from macrophage attack.^36^ Macrophages can be separated into two types, M1 and M2. M1 macrophages are responsible for recognizing and destroying tumour cells, while M2 macrophages exhibit tumour‐promoting properties by promoting angiogenesis, tissue remodelling and adaptive immunity suppression.^37^ Liu et al. injected mice with OSCC cells and infected tumour sites with a suspension of antibiotics‐inactivated *P. gingivalis*. As a result, they found *P. gingivalis* inhibits the phagocytosis of OSCC cells by M1 and induces functional polarization of macrophages into M2 tumour‐associated macrophages in mice models. Thus, an immunosuppressive tumour microenvironment is formed.^36^

### 2.3 In vitro evidence of periodontal pathogens influencing oral cancer

Inflammation‐related mechanisms play an important role in the involvement of *P. gingivalis* and *F. nucleatum* in carcinogenesis. For example, TLR signalling is activated in oral epithelial cells after infection by *P. gingivalis* and *F. nucleatum*. Increased expression of IL‐6 and STAT3 are observed, which in turn upregulates some important effectors, such as cyclin D1, matrix metalloproteinase (MMP) 9 and heparinase, promoting OSCC growth and invasiveness.^34^ Besides, *P. gingivalis* and its membrane can trigger NF‐κB and MAPK signalling pathways in malignant and primary human oral epithelial cells, which are downstream of TLR.^38^ Similar to *P. gingivalis*, a study found the number of NF‐κB nuclear translocations in oral epithelial cells, H400, was significantly increased following exposure to *F. nucleatum*.^39^ Gene expression analysis suggested that a number of transcripts regulated by the NF‐κB pathway, including tumour necrosis factor‐α (TNF‐α), monocyte chemoattractant protein‐1 (MCP‐1), GM‐CSF, interleukin‐1β (IL‐1β) and IL‐8, were upregulated after both *P. gingivalis* and *F. nucleatum* infection.^39^ Activation of these inflammation‐related mechanisms may lead to subsequent oxidative stress and cellular DNA damage, which are thought to be important processes in carcinogenesis.

Additionally, periodontal pathogens are able to facilitate the proliferation of tumour cells through different pathways. Some researchers found that *F. nucleatum* caused severe DNA damage such that intracellular Ku70 was insufficient to provide timely repair and the expression of wild p53 was limited. As a result, this dampened repair mechanism leads to abnormal proliferation of OSCC cells.^40^ Cyclin D1, a regulatory factor of cell proliferation, may lead to uncontrolled proliferation when it is overexpressed. Chang et al. reported *P. gingivalis* could continuously increase the level of cyclin D1 via the miR‐21/PDCD4/AP‐1 negative feedback pathway, thus promoting the proliferation of OSCC cells.^41^ Hoppe et al. also found that OSCC cells responded to the infection of *P. gingivalis* by upregulation of α‐defensin, resulting in a pro‐proliferative environment that promotes tumour growth.^42^ However, it is also worth mentioning that the effect of *P. gingivalis* on the biological behaviour of cancer cells remains controversial. Another research has found that *P. gingivalis* inhibited cell proliferation by inducing G1 arrest and autophagy in oral cancer cells.^43^ This may be related to the status of *P. gingivalis*, as well as the tissue specificity. Therefore, many factors should be considered carefully to determine the host cells' response to *P. gingivalis* infection.

**FIGURE 1:**
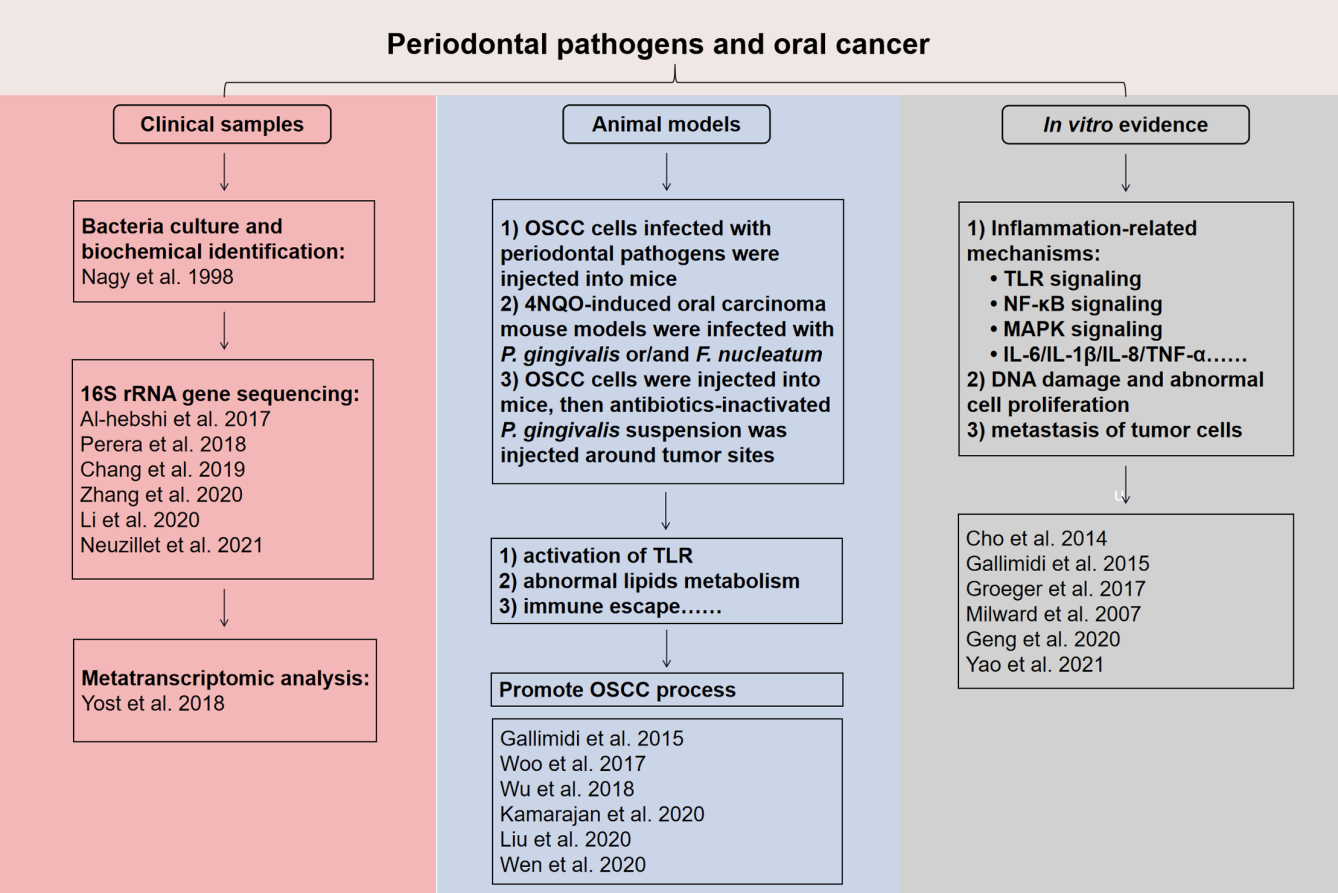
Summary of several methods for the study of periodontal pathogens‐driven oral cancer. To investigate the link between periodontal pathogens and oral cancer, human clinical samples were examined for microbial species and functional analysis. In addition, researchers established various animal models or performed in vitro cell experiments to confirm the effect of periodontal pathogens on oral cancer.^110^, ^111^, ^112^, ^113^

In addition to abnormal cell proliferation, bacteria also have an impact on the metastasis of tumour cells. It has been suggested that EMT stimulated by periodontal pathogens contributes to increasing metastasis of OSCC. Summarily, researchers demonstrated a strong association between periodontal pathogens and oral cancer through a variety of methods (Figure 1).

## 3 MECHANISMS THAT REGULATING EMT

Epithelial cells are one of the most ubiquitous cell types in the human body. It forms boundaries between different environments. The typical characteristic of all epithelial sheets is adherent junctions (AJs), which constitute apical adhesive structures.^44^ However, during the process of EMT, epithelial cells lose their representative cobblestone epithelial appearance and acquire a spindle‐shaped, mesenchymal morphology. At the molecular level, epithelial cells lose epithelial markers, such as epithelial cadherin (E‐cadherin), occludin and cytokeratins, which leads to the loss of cell–cell adhesion and apical‐basal polarity.^10^, ^11^, ^45^ Especially the expression of E‐cadherin, the main molecule of stable AJs, is repressed mostly and considered as a key event in EMT. By contrast, the cells start to express mesenchymal markers, notably vimentin, neural cadherin (N‐ cadherin) and fibronectin.^10^, ^45^ The entire process of EMT is regulated by various transcription factors, cytokines, small noncoding RNAs, et al. The following sections will introduce these mechanisms.

### 3.1 Transcription factors that induce EMT

There are different types of transcriptional regulators, including Snail, Slug, zinc finger E‐box‐binding homeobox (ZEB) 1/2, E12/E47 and Twist‐related protein (Twist) 1/2, which are collectively known as EMT‐inducing transcription factors (EMT‐TFs).^46^, ^47^ They can cooperate with several others, and form a complex network that mediates the cellular conversion during developmental and pathological processes.

Among vertebrates, Snail1 (Snail), Snail2 (Slug) and Snail3 (Smuc) are members of Snail family that have been identified.^48^ As mentioned above, one of the typical manifestations of EMT is the downregulation of E‐cadherin. Both Snail and Slug can bind to specific E boxes of the proximal E‐cadherin promoter, and Snail has a much higher affinity for E boxes than Slug.^49^ Snail recruit Sin3A/Histone Deacetylase 1 (HDAC1)/HDAC2 complex, which depends on the SNAG domain, to deacetylate histone H3 and H4 at E‐cadherin promoter.^49^ Furthermore, Snail is responsible for PRC2 recruitment to the CDH1 (coding for E‐cadherin) promoter and induce the subsequent trimethylation of H3K27, so that the expression of E‐cadherin is repressed.^50^ Slug also has the SNAG domain, which can interact with the corepressor NCoR to induce effective EMT. However, there is a SLUG domain within Slug that can recruit C‐terminal‐binding protein (CtBP), which plays a negative modulation of Slug‐mediated EMT. It is speculated that the SLUG domain of Slug provides a conformational state for Slug that hinders the recruitment of NCoR by the SNAG domain, resulting in subsequent E‐cadherin inhibition and EMT induction being affected. This may explain why the repression potency of Slug is lower than Snail.^51^ In addition, Snail and Slug act on other epithelial genes as well, such as the tight junction protein claudin1, by binding to proximal E‐boxes.^52^

The ZEB family and E12/E47 also directly decrease the expression of E‐cadherin. The ZEB family consists of ZEB1 and ZEB2, whose characteristic is the presence of a central homeodomain and two zinc finger clusters at each end.^53^ ZEB1/2 can simultaneously bind two zinc finger clusters to high‐affinity binding sites consisted of bipartite E2 boxes to downregulate mammalian E‐cadherin transcription.^53^, ^54^ Like the Snail family, ZEB also recruits specific chromatin‐remodelling complexes. CtBP is the corepressor recruited to E‐box by ZEB to regulate E‐cadherin expression.^15^, ^53^ In addition, BRG1, independent of CtBP, interacts with the N‐terminal region of ZEB1 to inhibit E‐cadherin.^55^ As for E12/E47, it not only acts as a repressor of E‐cadherin expression, but is also involved in the maintenance of EMT through its interaction with the Id1 protein.^56^, ^57^

Twist1/2 is commonly regarded as an indirect repressor of CDH1.^58^ The Twist is capable of recruiting the Mi2/nucleosome remodelling and deacetylase (Mi2/NuRD) complex to the E‐cadherin promoter region, to modify histones, remodel chromatin and repress transcription. Fu et al. found knocking down either MTA2 or RbAp46, components of the Mi2/NuRD complex, increased the E‐cadherin promoter activity in the same manner as knocking out Twist. This suggests that the Mi2/NuRD complex is essential for Twist‐mediated E‐cadherin inhibition.^59^ However, there is also a study with a different view that Twist can bind directly to the E‐boxes 2 and 3 on the CDH1 promoter, whereas Snail binds to E‐box 1.^60^

### 3.2 Cytokines and signalling pathways in EMT

EMT can be induced by several cytokines, such as transforming growth factor‐β (TGF‐β), TNF‐α, MCP‐1/CCL2, IL‐6 and signalling pathways including Wnt, Notch, NF‐κB, etc.^11^, ^47^ The efficient activation of signalling pathways is the crucial driving force of cytokines‐induced EMT. For instance, TGF‐β can activate TGF‐β family receptor signalling in a SMAD‐dependent manner to increase the expression of some EMT‐TFs, like Snail, ZEB and Twist. TGF‐β also induces EMT in a non‐SMAD‐dependent way, like ERK, MARK, NF‐κB, TRAF6/TAK1/JNK/P38 signalling and PI3K/AKT/mTOR signalling.^61^, ^62^

NF‐κB pathway participates in TNF‐α‐induced EMT as well. TNF‐α rapidly activates NF‐κB through two classical modules of NF‐κB signalling, IKKβ and p65, and upregulates Twist1 expression. More specifically, TNF‐α triggers IKKβ, leading to nuclear translocation and activation of p65, which subsequently binds to the Twist promoter to regulate its transcription.^63^ It is worth mentioning that TNF‐α and TGF‐β1 synergistically enhance the activation of many signalling pathways by activating TAK1, thus promoting EMT.^62^ Besides, TNF‐α is able to promote TGF‐β receptor expression.^62^ A study shows that TNF‐α could enhance TGF‐β‐induced endothelial‐to‐mesenchymal transition (EndMT), a subcategory of EMT, by augmenting TGF‐β family signals, which was supported by the elevated expression of TGF‐β type I receptor, TGF‐β2, integrin αv and activin A.^64^

CCL2, cooperating with its receptor CCR2, activates the Hedgehog signalling pathway, resulting in the upregulation of Snail and EMT.^65^ IL‐6 promotes EMT via the Wnt/β‐catenin pathway in STAT3/ERK‐dependent manner.^66^ Apart from the above pathways, EMT is regulated by an intricate network of cross‐signalling involving transcription factors, cytokines, growth factors and signalling pathways.

### 3.3 MicroRNAs in EMT

MicroRNAs (miRNAs) are small endogenous RNAs that regulate target genes silencing post‐transcriptionally.^67^ They can activate or inhibit EMT‐TFs and related signalling pathways, to control the EMT process. For example, the miR‐200 family, an acknowledged inhibitor of EMT, markedly suppresses the expression of ZEB1 and then prevents EMT. In turn, ZEB1 can downregulate the transcription of miR‐200. Thus, the two form a double negative feedback loop that maintain a dynamic balance between epithelial and mesenchymal states.^68^, ^69^ Overexpressed miR‐199a‐5p inhibits EMT by targeting ZEB1 via PI3K/AKT/mTOR signalling.^70^ MiR‐26a and miR‐26b act on the target gene Jagged‐1 and negatively regulate Jagged‐1/Notch signalling and ultimately suppress EMT.^71^ Exosomal miR‐92a‐3p promotes cell stemness and EMT through decreasing the expression of FBXW7 and MOAP1 and activating the Wnt/β‐catenin pathway.^72^ In addition, it was mentioned that miR‐7, miR‐100 and miR‐125 were related to the release of inflammatory cytokines and metalloproteases in gingival fibroblasts during the early stages of periodontitis. It was supposed some miRNAs might influence the occurrence and development of oral cancer through inflammatory response in addition to the EMT process.^73^

### 3.4 Other long noncoding RNAs in EMT

Long noncoding RNAs (lncRNAs), although they do not encode proteins themselves, can regulate the expression of protein‐coding genes by recruiting or sequestrating gene‐regulatory proteins and miRNAs. Recent evidence has proved that lncRNAs take part in EMT mainly by interacting with the master regulator of EMT.^74^ They can activate EMT‐TFs or signalling pathways like TGF‐β, and can also act as competitive endogenous RNAs (ceRNAs) for miRNAs to control EMT processes. For instance, lncRNA, metastasis‐associated lung adenocarcinoma transcript (MALAT1), is involved in TGF‐β1‐mediated EMT by significantly increasing ZEB1 expression in diabetic wounds.^75^ In oesophageal cancer, MALAT1 activates the Ezh2‐Notch1 signalling pathway to promote EMT.^76^ MALAT1 upregulates TGF‐β and diminishes E‐cadherin by acting as a ceRNA to miR‐101 in colorectal cancer,^77^ and targets ZEB2 by competing with miR‐204 in breast cancer.^78^ All of these findings suggest that lncRNAs are involved in EMT through multiple pathways.

## 4 *P. GINGIVALIS* INDUCES EMT

### 4.1 *P. gingivalis*‐induced EMT in oral cancer

*P. gingivalis* is a gram‐negative anaerobic bacterium which is widely regarded as a ‘keystone’ periodontopathogen and emerging oncopathogen. *P. gingivalis* produces a variety of virulence factors, including lipopolysaccharide (LPS), gingipains, fimbriae/pili, exopolysaccharides, proteolytic enzymes, kinases and phosphatases, to evade the human immune defence system and destroy periodontal tissues.^79^, ^80^ With the help of cell surface fimbriae (FimA), *P. gingivalis* colonizes and invades epithelial cells.^81^ To simulate chronic oral irritation caused by *P. gingivalis*, Ha et al. infected OSCC cells with *P. gingivalis* twice a week for a total of 5 weeks.^82^ They detected a decrease of epithelial cell markers with concomitant increase of mesenchymal markers.^82^ This is the first time that *P. gingivalis* has been shown to trigger EMT‐like changes in oral cancer cells in vitro. CD44 and CD133 are well known hallmarks of stemness.^83^ After *P. gingivalis*‐induced EMT, the expression of both CD44 and CD133 in oral cancer cells were significantly increased, suggesting these cells have acquired stemness and tumour sphere‐forming ability.^82^ Epithelial cells undergoing EMT become more aggressive, for they gain the ability of invasion and migration.^82^ MMP can degrade extracellular matrix and basement components to motivate invasion and potential metastasis of cancer cells.^84^ Numerous studies have confirmed *P. gingivalis* enhances the release of MMPs in OSCC cells, including MMP‐1, −2, −7, −9 and − 10,^82^, ^85^, ^86^ which is also consistent with the cellular characteristics undergoing EMT.

### 4.2 Mechanisms of *P. gingivalis*‐induced EMT

As previously discussed, there are an array of EMT‐TFs controlling the process of EMT. It has been observed that the protein levels of Snail and Slug were significantly increased in *P. gingivalis*‐infected cells.^86^ Simultaneously, in another study, both Snail and Twist were increased 6‐fold after 8 d of culture following *P. gingivalis* stimulation.^9^ Sztukowska et al. found strains of *P. gingivalis* normally expressed FimA could enhance ZEB1 mRNA levels in gingival epithelial cells while strain W83, which lacked the FimA protein, made no difference to ZEB1 levels.^87^ That indicates FimA may be a key effector protein for ZEB1 induction. In addition to in vitro evidence, the induction of ZEB1 by *P. gingivalis* was also confirmed in vivo situations using mouse models.^87^

Glycogen synthase kinase 3 beta (GSK3β) is a regulator of Snail, Slug and ZEB1 transcription factors. *P. gingivalis* can increase PI3K/Akt pathway activation,^88^, ^89^ which results in the phosphorylation of GSK3β and the subsequent upregulation of Snail, Slug and ZEB1.^86^ These molecular changes firstly facilitate the loss of E‐cadherin and noncanonical activation of β‐catenin, then the change of subcellular localization of β‐catenin induces the increased expression of mesenchymal markers vimentin and MMP‐2, −7 and −9.^86^ *P. gingivalis* is also able to induce extracellular secretion of TGF‐β1, another regulator that can activate protein kinase Akt.^90^ Moreover, low molecular weight tyrosine phosphatase (Ltp1) is secreted and delivered by *P. gingivalis* in epithelial cells. It can dephosphorylate PTEN, a negative regulator of Akt signalling, and eventually cause proteasomal degradation.^91^ Decreased PTEN activity mitigates the inhibition of PI3K/Akt pathway. Thus, the production of regulator of growth and cell cycle is increased, leading to the upregulation of ZEB2 and a partial mesenchymal phenotype in epithelial cells.^91^

Additionally, there are many cytokines playing an important role in *P. gingivalis*‐induced EMT. Abdulkareem et al. investigated the levels of several cytokines involving TGF‐β1, TNF‐α and EGF in OSCC cells infected by *P. gingivalis* and detected a significant increase.^9^ These three cytokines stabilize and activate Snail via a common EMT‐signalling pathway.^9^ Notably, compared to day 8, *P. gingivalis* infection did not induce an obvious increase in the expression of all these cytokines on day 1, suggesting that EMT requires a long‐lasting stimulus by *P. gingivalis*.^9^ Besides, IL‐8 takes a part in the occurrence of EMT and its maintenance, and a markedly increased expression of IL‐8 was monitored in *P. gingivalis*‐infected OSCC cells.^82^

## 5 *F. NUCLEATUM* INDUCES EMT

*F. nucleatum* has long been regarded as an opportunistic pathogen and been commonly found in oral cavity and other mucosal sites. *F. nucleatum* was frequently isolated and identified in various anaerobic samples from patients with different infections, not only periodontal disease.^92^ In recent years, more attention has been paid to the biological role of *F. nucleatum* in enhancing oral cancer progression. Similar to *P. gingivalis*, *F. nucleatum* infection of oral cancer cells can increase the expression of various EMT‐associated transcription factors and cytokines, including ZEB1, IL‐8, TGF‐β1, EGF and TNF‐α.^9^, ^93^ Harrandah et al. found *F. nucleatum* culture supernatant component alone was sufficient to induce IL‐8 overexpression, which implied that *F. nucleatum* could promote cancer without direct contact with tumour cells.^93^ They further investigated LPS/TLR4 pathway was one of pathways through which *F. nucleatum* interact with oral cancer cells and induce IL‐8 secretion.^93^ Interestingly, Abdulkareem et al. reached similar conclusions in primary oral keratinocytes from rats, a nontumour cell, that is, *F. nucleatum* could promote EMT through LPS/TLR4 pathway.^94^ Furthermore, *F. nucleatum* upregulates the expression of miR4435‐2HG in both non‐neoplastic and neoplastic oral epithelial cells.^95^ MiR4435‐2HG can downregulate miR‐296‐5p levels, leading to the overexpression of its target gene Akt2, and finally activate Snai1 that drive the progression of EMT.^95^ In addition to studies on the pathway of *F. nucleatum*‐induced EMT, some researchers found *F. nucleatum* infection altered partial‐EMT‐related gene expression, such as SERPINE1, CDH13, ITGA5, TGFBI, LAMC218 and P4HA2, which are also essential indicators of poor prognosis in head and neck squamous cell carcinoma.^96^

*F. nucleatum* has long been recognized as one of the promoting factors for colorectal cancer (CRC). There are many studies on how *F. nucleatum* promotes EMT in CRC. For instance, Rubinstein et al. found Fusobacterium adhesin A (FadA), a virulence factor identified from *F. nucleatum*, can directly bind to E‐cadherin on CRC cells and activate β‐catenin, contributing to the overexpression of Wnt signalling.^97^ Wnt target genes include transcription factors ZEB1 and Snai1.^98^ Additionally, another study discovered Annexin A1, a novel Wnt/β‐catenin pathway modulator, can be induced by the combination of FadA and E‐cadherin and thus activate β‐catenin.^99^ *F. nucleatum* also accelerates the EMT process through other signalling pathway, such as EGFR, STAT3 and TLR4/AKT/NRF2 signalling pathway.^100^, ^101^, ^102^

## 6 COMMUNITY ACTION OF PERIODONTAL PATHOGENS IN THE REGULATION OF EMT

The preceding text describes the carcinogenic effects of *P. gingivalis* and *F. nucleatum* as individual species. However, these studies have some limitations, ignoring the community nature of periodontal pathogens infection. Bacteria often exist as a diverse and complex microbial community in oral cancer. In reality, bacteria have intricate social lives. They form tight biofilms in which they communicate with each other and exchange nutritional substrates, exhibit division of labor, compete and cooperate.^103^ Because of the division of functions among community participants, they are codependent with each other. So, the community emerges as the pathogenic unit, instead of individual species.^103^ This is consistent with studies on the relationship between microbiotas and OSCC in different countries, showing that these communities are similar in function, rather than composition.^104^ So, the question currently arises as to how other species in the community potentially influence periodontal pathogens infections. Therefore, it is necessary to examine multiple mechanisms of polymicrobial synergy and antagonism in oral cancer.

*P. gingivalis* forms in a synergistic community with *Streptococcus gordonii* (*S. gordonii*) and *F. nucleatum*. Although neither *S. gordonii* nor *F. nucleatum* is able to modulate ZEB1 mRNA levels, combinations of *P. gingivalis* with both two species still increase ZEB1 expression,^87^ indicating these microbial communities may contribute to EMT. Interestingly, another research pointed out that *S. gordonii* was able to antagonize the ZEB2 induction by *P. gingivalis* though it did not directly regulate ZEB2.^105^ *P. gingivalis* controls the induction of ZEB2 by activating the FOXO1 transcription factor via dephosphorylation of the serine 256 and serine 329 residues in FOXO1. However, *S. gordonii* blocks the activation of FOXO1 through TAK1‐NLK negative regulatory pathway.^105^ Moreover, olfactomedin 4 (OLFM4) was newly found to be differently regulated by *P. gingivalis* and its gingipain proteases.^106^ OLFM4 is a potential biomarker for head and neck squamous cell carcinomas. It accumulates in the secretome of this disease and takes part in the regulation of EMT.^107^, ^108^ *P. gingivalis* controls OLFM4 by activating its upstream Notch1/Jagged1 signalling cascade. After Notch1 binds to Jagged1, *P. gingivalis* gingipain proteases cleave the extracellular domain of Notch1 to activate signalling. However, *S. gordonii* inactivates the gingipains of *P. gingivalis* by hydrogen peroxide, thus antagonizing the Notch signalling.^106^

## 7 CONCLUSION

**FIGURE 2:**
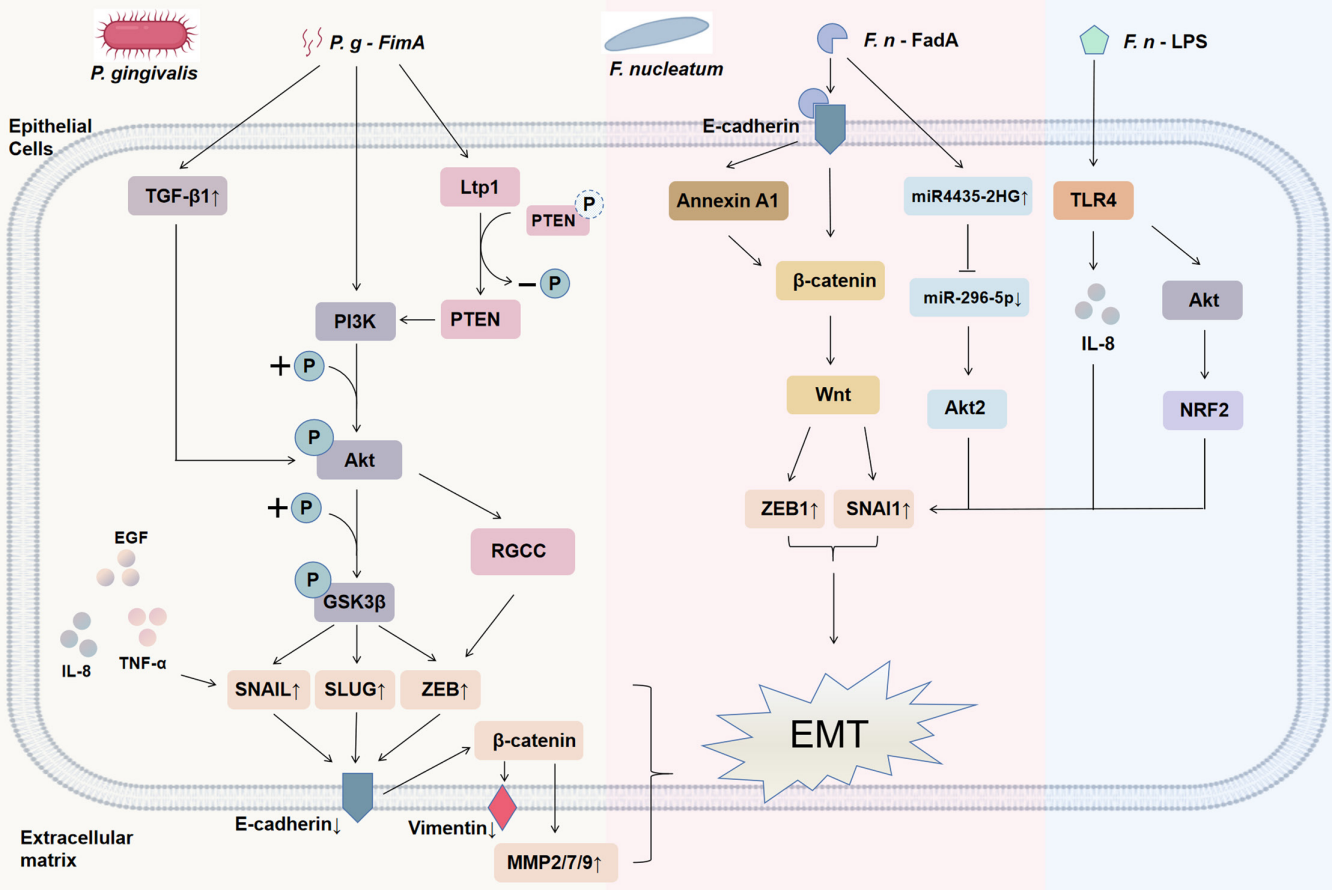
Mechanisms of *P. gingivalis*/*F. nucleatum*‐induced EMT.

As a multifactorial disease, cancer is caused by complex interactions between genetic, environmental and other factors. So far, periodontal pathogens cannot be considered as a direct aetiology of oral cancer, but there is no doubt that periodontal pathogens have a strong link with oral cancer. Elevated levels of some oral miRNAs in patients with periodontitis, as well as the release of some systemic inflammatory markers, like NT‐proBNP, have been shown to affect endothelial function and may also drive the progression of oral cancer.^73^, ^109^ EMT mediated by periodontal pathogens is widely recognized for promoting the malignant metastasis of tumour cells and the formation of secondary tumours. This review introduced a wealth of evidence for EMT caused by *P. gingivalis* and *F. nucleatum* in oral cancer progression (Figure 2), but our current appreciation of the involvement of oral bacteria in cancer through EMT may be just the tip of the iceberg. Most researchers still only study the role of bacteria as a single species, ignoring that the contribution of bacteria in oral cancer may be based on the community as the pathogenic unit. In particular, the literature on the changes in the abundance of microbial species in oral cancer is rather provocative but inconclusive. Thus, it is necessary to further explore the association between the microbiota and oral cancer at functional levels. Then, the nature of the role of periodontal pathogens requires further study. Large‐scale prospective cohort studies are lacking at this stage, so causality cannot be established.

In summary, periodontal pathogens‐induced EMT is one of the vital links in the process of oral cancer, which requires a more in‐depth understanding of the complex molecular pathways and its multiple regulatory molecules. Certainly, our ultimate goal is to use it as a biomarker for progression and recurrence, and as a novel therapeutic target for oral cancer. Nevertheless, more studies are needed including animal models and clinical trials.

## AUTHOR CONTRIBUTIONS

**Yiwei Ma:** Conceptualization (lead); data curation (lead); investigation (lead); writing – original draft (lead). **Yingyi Yu:** Investigation (supporting). **Yuqing Yin:** Investigation (supporting). **Liu Wang:** Investigation (supporting). **Huishun Yang:** Investigation (supporting). **Qifan Zheng:** Investigation (supporting). **Shiyin Luo:** Investigation (supporting). **Yaping Pan:** Supervision (equal); writing – review and editing (equal). **Dongmei Zhang:** Funding acquisition (equal); project administration (equal); supervision (equal); writing – review and editing (equal).

## FUNDING INFORMATION

National Natural Science Foundation of China NO.81970943.

## CONFLICT OF INTEREST STATEMENT

The authors confirm that there are no conflicts of interest.

## Data Availability

Data sharing not applicable to this article as no datasets were generated or analysed during the current study.
